# Ecological complexity and the biosphere: the next 30 years

**DOI:** 10.1098/rstb.2021.0376

**Published:** 2022-08-15

**Authors:** Ricard Solé, Simon Levin

**Affiliations:** ^1^ ICREA-Complex Systems Lab, CSIC Universitat Pompeu Fabra, Dr Aiguader 80, Barcelona 08003, Spain; ^2^ CSIC-UPF, Institut de Biologia Evolutiva, Pg Maritim de la Barceloneta 37, Barcelona 08003, Spain; ^3^ Santa Fe Institute, 1399 Hyde Park Road, Santa Fe, NM 87501, USA; ^4^ Department of Ecology and Evolutionary Biology, Princeton University, Princeton, NJ 08544, USA

**Keywords:** ecological networks, restoration, climate change, bioengineering, tipping points, biodiversity

## Abstract

Global warming, habitat loss and overexploitation of limited resources are leading to alarming biodiversity declines. Ecosystems are complex adaptive systems that display multiple alternative states and can shift from one to another in abrupt ways. Some of these tipping points have been identified and predicted by mathematical and computational models. Moreover, multiple scales are involved and potential mitigation or intervention scenarios are tied to particular levels of complexity, from cells to human–environment coupled systems. In dealing with a biosphere where humans are part of a complex, endangered ecological network, novel theoretical and engineering approaches need to be considered. At the centre of most research efforts is biodiversity, which is essential to maintain community resilience and ecosystem services. What can be done to mitigate, counterbalance or prevent tipping points? Using a 30-year window, we explore recent approaches to sense, preserve and restore ecosystem resilience as well as a number of proposed interventions (from afforestation to bioengineering) directed to mitigate or reverse ecosystem collapse. The year 2050 is taken as a representative future horizon that combines a time scale where deep ecological changes will occur and proposed solutions might be effective.

This article is part of the theme issue ‘Ecological complexity and the biosphere: the next 30 years’.

*The future cannot be predicted, but futures can be invented*.

Denis Gabor

## 1. Introduction

**Figure 1.**
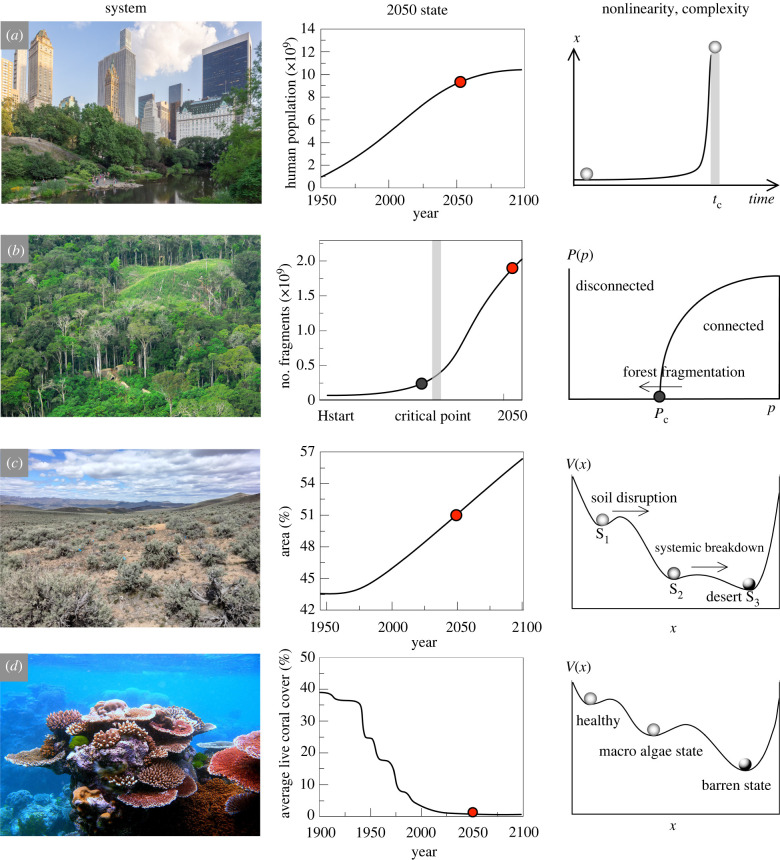
Ecological complexity challenges for 2050. With the rise of global temperatures, population growth and the resulting pressure on resources and habitats, biodiversity will face major threats. One crucial role of science is to develop reliable predictions of future trends. Here, four examples are chosen (left) along with current forecasts (central column, estimated 2050 states indicated with a red circle) and examples of the complex systems approaches used (right). (*a*) Urban centres (image of Central Park, New York, by Ajay Suresh, Creative Commons) are rapidly expanding as massive migrations occur towards cities. Human population growth (centre) is slowly decelerating, but two extra billion humans will be added to the current numbers, reaching 9.7 billion by 2050. The current trend is a consequence of the nonlinearities associated with hyperbolic dynamics, which predicts a singularity at a given finite time *t*_*c*_ (right). (*b*) Rainforests (left image by Gleilson Miranda, Creative Commons) are experiencing rapid loss and fragmentation of their habitats, with predicted critical points (centre plot, grey bar, see [2]) to be reached in a few decades. These critical points correspond to percolation thresholds (right panel). (*c*) Drylands (image courtesy of David Huber) are expanding and will grow from the current 40% to more than 50% in just three decades. Models of drylands involving vegetation cover as a key variable predict sharp transitions between alternative states, connected through three different shifts [3]. Here two of them are indicated. (*d*) Marine ecosystems, and coral reefs (left image by Toby Hudson, Creative Commmons) in particular, are being affected by warming ocean temperatures, eutrophication, pathogens and overfishing. Reef cover is rapidly shrinking and might experience massive decays in the next decades. Here, the previous and predicted time series of coral reef cover in Hawaii is shown (centre, data from https://19january2017snapshot.epa.gov/cira/climate-action-benefits-coral-reefs_.html). Multiple alternative states have been identified (right) with different sources of stress causing jumps from one state to another.

Over the last decades, a general consensus among scientists from very diverse disciplines has been emerging about the future of our planet and our society (Intergovernmental Panel on Climate Change) and provides a grim picture of how global warming will affect the biosphere in multiple ways and across scales [1]. Regional, continental and planetary-scale changes are taking place at an accelerated pace. Greenhouse gases are the most obvious example of such a trend, with CO_2_ in particular displaying a fast increase that has no equivalent over the past 500 Myr. This rise is a consequence of industrialization and the parallel population growth, particularly in urban areas (figure 1*a*). By 2050, 70% of humankind will live in cities. Despite the deceleration of this process (largely due to reduced fertility rates and changes in women’s status), the predicted expansion gives a staggering 9.7 billion people. The ultimate reason for this explosive growth has to be found in the mathematics of population dynamics. The historical record of modelling in climate science and conservation studies starts long ago. In many cases, predicted outcomes were tied to theory (either mathematical or computational) that would help quantify future scenarios of change, decay and recovery [4,5]. A common goal (and a nontrivial problem) in all these approaches is prediction.

Historical examples of long-term prediction include the famous 1972s *The limits to growth* report that was intended to present the first long-term simulation of economic and population growth [6]. It involved a simplified description of human systems and their interactions with a world with finite resources. The model efforts, led by Donella Meadows, incorporated several key variables known to grow with time, including human population, food production, industrialization, pollution and consumption of non-renewable natural resources. The methodology was inspired by the work of Jay Forrester [7]. He was a pioneer of so-called Systems Science, a field that takes a complexity view of the world where interactions among many components are treated as simplified, deterministic dynamical systems.

The report was cautious about the assumptions and its potential implications: ‘The model we have constructed is, like every other model, imperfect, oversimplified and unfinished’ [6]. One of the key predictions made by the report is described as follows (pp. 23–24):

If the present growth trends in world population, industrialization, pollution, food production and resource depletion continue unchanged, the limits to growth on this planet will be reached sometime within the next one hundred years. The most probable result will be a rather sudden and uncontrollable decline in both population and industrial capacity.

Despite all the unknowns, the crucial outcome of the report was clear. Business as usual in a planet with limited resources and a rapidly (exponentially) expanding human population can only end up in unsustainable growth and collapse. A second message from the report sounds familiar nowadays: ‘the trends depicted above could be modified provided that sustainable growth is introduced, in such as way that rational use of resources allows the maintainance of stability while the basic material needs of each person on earth are satisfied and each person has an equal opportunity to realize his individual human potential’ [6, p. 24].

An obvious limitation of this kind of study is the requirement of model simplifications, such as ignoring geography or different sources of fluctuations, along with the inevitable limitations associated with parameter estimation. Most importantly, the use of a small number of variables seems inappropriate when trying to represent the complexity of the real world. The goal was to examine the interactions between the five variables within a two-century window (1900–2100). It thus includes past information that was used to calibrate some of the required parameters. In this way, Meadows’ model became the first integrated global model and inspired a great deal of studies since [8].

Nowadays, any realistic assessment of the future of the planet requires consideration of the explicit role played by climate. As global warming and an intensive exploitation of planet resources keep rapidly increasing, the analysis of past climates and modelling efforts suggest that future changes can unfold in potentially catastrophic ways [9]. As far-from-equilibrium, dissipative structures, ecological systems exhibit nonlinear dynamical properties that pervade their stability but are also responsible for their fragility under stress. They are in fact *complex adaptive systems* (CAS) [10]. Crucial features of CAS include spatial and temporal heterogeneity, diversity and nonlinearity [3]. It is in this context that integrative approaches to climate and the biosphere are of fundamental relevance.

Wide weather fluctuations, alarming biodiversity declines and social unrest are already here. Future potential tipping points have been identified, while most predicted climate change scenarios seem confirmed and consistent with worst-case outcomes. What can be done to reverse, counterbalance or prevent tipping points? Many different proposals have been suggested based on sustainable growth, restoration strategies and increased clean energy use. But the time scale for effective measures is rapidly shrinking. Confronted with a planet decline where humans are part of a complex, endangered ecological network, novel approaches need to be taken. All these approaches include unsolved, multiscale problems and will need to be applied in a social context dominated by cities, political instability and rising inequality. A complex systems perspective including all key aspects of the problem is required, pointing to an agenda of well-defined alternatives.

What are the challenges ahead for the next decades? Using 2050 as a potential time horizon, here we summarize some of the key issues associated with the future of the biosphere under a complex systems perspective. Much has taken place since the publication of *The* *limits to growth* and the use of models is nowadays widespread. How can humans be included as part of modelling efforts? What kind of information is required to feed these models? What can be safely predicted? Answering these and other fundamental questions was the goal of a workshop hosted by the Santa Fe Institute in 2021. The meeting convened a group of researchers from diverse fields, from theoretical and conservation ecology to synthetic biology. This Theme Issue summarizes several key concepts associated with the nonlinear, complex nature of our biosphere and how these nonlinearities affect future trends. But the year 2050 needs to be seen also as a window to plan for interventions: what can be done from conservation, restoration and engineering?

## 2. Humans, defaunation and extinction

Extinction and biodiversity declines are two major consequences of the human-caused environmental crisis. Species loss has been accelerating at unprecedented levels. It is estimated that the current rate of species extinction is three orders of magnitude larger than the so-called background rate [11–13]. Because of an accelerated rate and the expected consequences for biodiversity, it is often said that we are entering the ‘sixth’ mass extinction [14,15]. The term Sixth Extinction was first coined in Leakey & Lewin [16] and refers to the previous five, well-established mass extinction events [17–22].

Along with extinction, Anthropocene defaunation has also been accelerating in both marine [23] and terrestrial [24] habitats. Vertebrate population abundances have experienced 25% average loss [25] and the numbers double when dealing with some invertebrate species. The latter trend has raised concerns in relation to the dramatic losses of insects that have taken place within the last few decades. As pointed out in [26], insect declines are particularly troubling, given the role played by them within ecological food webs. In general, the projected effects of climate change suggest that a massive biodiversity loss is on the horizon. This has been shown in a recent, systematic analysis of 30 000 marine and terrestrial species [27]. In particular, the study reveals that by 2050 the vast majority of these species will be exposed to abrupt changes.

What is the underlying force causing these rapid shifts? The answer of course needs to be found ultimately in the faster-than-exponential growth of our species in the last two centuries after a long period of time displaying no significant growth. A general argument (see [28] and references therein) to obtain hyperbolic growth goes as follows. The population *x*(*t*) changes in time can be described by an equation

2.1 $$\frac{dx}{dt}=rx(t)(K(t)-x(t)),$$

where there is a carrying capacity *K*(*t*) that increases with population due to the presence of innovations. Specifically, the interaction between population size and the potential for further expansion is assumed to take the general scaling form *K*(*t*) ∼ *x*^*γ*^ where *γ* = 1 would indicate a linear dependence between innovation and population and *γ* > 1 a *superlinear* scaling that seems characteristic of urban centres [29,30]. Cohen *et al*. [31] suggest that the carrying capacity *K*(*t*) increases with *x*(*t*) due to constant technological, medical and energy use improvements as well as the expansion of human populations into new habitats. If this coupling is such that eventually *K*(*t*) > *x*(*t*), we have growth, and (asymptotically) the shape of this growth is obtained by solving the corresponding equation

2.2 $$\frac{dx}{dt}=r{\left(x(t)\right)}^{1+\gamma },$$

which implies that growth rate accelerates as *r*(*x*(*t*))^1+*γ*^ [28,29]. When this equation is solved, the nature of the nonlinearity reveals itself: a singularity is obtained for a finite time (figure 1*a*, right) as shown by the solution of the previous equation, which gives

2.3 $$x(t)=C{\left(\frac{1}{t_{c}-t}\right)}^{1/\gamma },$$

where *C* is a constant. This solution predicts that *x* → ∞ (a faster-than-exponential divergence) for a given *finite* time $t=t_{c}=1/\gamma rx_{o}^{\gamma }$ (with *x*_0_ indicating the initial population). The consequences of this nonlinear behaviour and the superlinear coupling between innovation and population size is that, in general, crises are expected to occur [29,32].

As discussed in Raven [21], the shrinking of biodiversity is deeply connected to the massive expansion of agriculture and domestication of animals, which fostered a hyperexponential growth. In the language of complex systems, agriculture represents a major transition: the emergence of ultra society [33,34], which allowed us to reduce environmental uncertainty. In this case, unfortunately, the ‘population bomb’ is also an inevitable result of the positive feedbacks associated with an innovation-driven growth [21].

The main threats are thus associated with overexploitation and agriculture. Species loss is a consequence of human development and the intensification of land use. How can this be changed? As pointed out in Dirzo *et al.* [35], biodiversity declines are the result of the intersection of two CAS: ecosystem functioning and human culture. To approach the main problem, these authors suggest reducing the scale of the human enterprise. That would include reduction of birth rates along with inequality and excess waste production, but also increasing collective awareness [35]. The problem of how to deal with this in the next decades is strongly tied to the ways in which innovation and demography interact. Recent efforts have addressed this in mathematical terms by exploring where technological innovations are driven by cumulative cultural evolution [36]. Perhaps not surprisingly, this work claims that there is room for the human population to grow without exhausting ecosystem services, but this can only occur under some given, well-defined conditions. They conclude that ‘The only way to fill the planet with humans under this scenario of negative technologies is by reducing the technological stock to a minimum. Otherwise, the only feasible equilibrium is associated with population collapse’ [36, p. 1].

A conservationist approach to species loss and defaunation calls for a large-scale, planetary effort aimed at the conservation of wild habitats. This concept was popularized by the late E. O. Wilson as the ‘Half Earth’ proposal [37]. In a nutshell, by setting aside half of extant (both marine and terrestrial) wild habitats, a very ambitious agenda has been under discussion since its suggestion in 2016. Protecting such a gigantic area is of course plagued with all kinds of obstacles and constraints. The idea is appealing because of its simplicity, but there are pressing issues regarding the politics, economic and social requirements on the level of management and governance [38]. Planning towards a 2050 horizon has led to a ‘Global Deal for Nature’ and specific assessment has been defined [39,40]. However, it is hard to plan in this direction given the fact that crops, settlements and forestry already cover 57% of emerged lands. In this context, the great challenge here is how to make compatible—within the 2050 time scale—expanding protected habitats in a world dominated by agriculture. In this context, some studies suggest that protection should address high-biodiversity areas involving small-ranged species [41].

In comparison with cultivated lands and areas used by livestock grazing (close to 25%), cities and other infrastructures only cover around 2%. And yet, population growth will occur mainly in an urban context and biodiversity loss has been actually shown to be tied to urban land conversions [42]. Projected impacts to 2050 predict that unmitigated urbanization will jeopardize the survival of thousands of species whose habitats will be affected. Given the large energy requirements associated with economic growth and development, macroevolutionary models indicate that large amounts of energy will be needed to fuel economic growth [43]. Projected global energy consumption for 2050 shows that a vast increase in energy supply will be needed to meet the demands of projected population growth while lifting the developing world out of poverty and simultaneously maintaining the current standards of living in the most developed countries.

## 3. Resilience, networks and tipping points

Future changes in ecosystems under Anthropocenic driving forces are likely to be nonlinear. Nonlinear responses are a common property of all CAS (from biology and ecology to social and economic systems). These systems are often characterized by the presence of multiple alternative states, and in many cases transitions from one state to another are expected to be abrupt [9,44]. The importance of multiple attractors and their role in understanding the stability and resilience of ecosystems was early highlighted by Holling [45]. He and others stressed the relevance of these alternative states as an indicator that natural communities can display different stable patterns of organization and that transitions from one to another can involve a loss of resilience or even the collapse of the ecosystem. In its simplest form, the state of an ecological system is indicated by a single variable *x* (vegetation cover, population size) and described by means of a one-dimensional model

3.1 $$\frac{dx}{dt}=f_{\mu }(x)+\xi (t),$$

where *μ* indicates the presence of one or several parameters influencing the dynamical state. The set of different attractors (e.g. stable fixed points) is obtained from the equilibrium condition d*x*/d*t* = 0. In CAS, the function *f*_*μ*_(*x*) is nonlinear and the nature of the nonlinearities deeply influences the number and properties of the observable stable states. The last term in the right-hand side introduces additive noise.^1^

An example of nonlinear model showing tipping points is illustrated by this facilitation growth model under habitat loss

3.2 $$\frac{dx}{dt}=\mu x^{2}(1-D-x)-\delta x,$$

where a given population grows under a cooperative interaction with other members of its own species (encapsulated in *x*^2^) while growth is limited by the actual population size and the amount of habitat *D* that has been destroyed (with a corresponding saturation 1 − *D* − *x*). The extra term −*δx* stands for local extinction due to environmental stochasticity. The model exhibits a rich dynamical behaviour, including a catastrophic shift when habitat loss achieves a given threshold [47]. How good is this simple approximation? Can simple models help to get accurate understanding of real ecosystems? The answer is positive, and indeed low-dimensional models have been successful in making sense of ecological nonlinearities. Importantly, although more accurate approaches can be used to describe the same phenomena, all of them predict the presence of marked phases separated by sharp phase transition boundaries. The origin of such consistency is grounded in universal properties of complex systems [48].

In figure 1*b*–*d*, we give three well-known examples of systems that can display abrupt transitions and illustrate the power of a complex systems view, all of them sharing potential shifts to occur within our 2050 window. The first (figure 1*b*) is connected with the future fate of tropical forests under the current trends of habitat fragmentation [2]. Under a no-mitigation scenario, it was estimated that a dangerous threshold of fragmentation can be crossed in a few decades (figure 1*b*, centre). This is known as a *percolation* point and defines a critical transition that separates a connected from a disconnected system. The sharpness of this point has been known for a long time within the context of statistical physics [49–51]. Specifically, if we think about a given two-dimensional habitat in terms of a square lattice, and if *p* is the probability that a site is occupied by a tree (otherwise it would be empty) the theory predicts that a phase transition occurs at a given *p*_*c*_ (figure 1*b*, right). If we indicate as *P*(*p*) the fraction of sites belonging to the largest cluster we move from zero to a finite, significant value once we cross *p*_*c*_. Close to this point, a universal statistical pattern exists [52]: the relative frequency *P*(*s*) of finding a vegetation cluster of size *s* and (this can be the number of pixels from a remote sensing dataset) scales as a power law

3.3 $$P(s)∼s^{-\gamma },$$

where the exponent displays a universal value, namely *γ* ≈ 2 that is exactly what is found in tropical forest field data, thus indicating that the disconnection transition might not be far away. As a larger fraction of habitat is degraded or destroyed, isolated clusters of trees become more common. With increasing habitat loss, the number of forest fragments grows but most of the system remains connected through some path, until the density of trees moves below *p*_*c*_. At this point, the disconnected nature of the resulting forest landscape can effectively reduce the viability of local metapopulations and trigger extinction events [53,54]. This transition allows definition of scales in fragmented landscapes [55,56], definition of conservation strategies based on reconnecting fragments [57] and has also been connected to the impacts of expanded agricultural systems on sustainability [58].

Both the statistical patterns associated with percolation as well as the dynamical description given by nonlinear mathematical models combine to describe ecological complexity. In general, one of the goals of modelling ecosystems is to identify the potential repertoire of the alternative states associated with transition-like phenomena. The two next examples shown in figure 1*c*,*d* illustrate this. The first is given by the expansion of drylands and the second the rapid decline of coral reef ecosystems. In relation to the former, while increasing aridity will keep pushing the surface of arid and semiarid lands above 50% in 2050 (figure 1*c*, centre), it has been predicted that abrupt thresholds will be crossed by global drylands during this century [3,59,60]. The different states and the potential shifts from one to another can be represented as marbles in a mathematical landscape V(x) made of several valleys.^2^ In this case, three phases of change have been identified and associated with three well-defined levels of aridity. They correspond to changes in vegetation composition, structural loses associated with decay in fertility and microbiome quality, and a final loss of diversity and plant cover leading to a desert state [3,63]. These studies provide the basis for forecast. Specifically, although no explicit time-dependent behaviour is used, one can take advantage of the so-called ergodic behaviour: the global-level statistical sample is a snapshot that captures many different local transitions. In other words, the fact that vegetation cover *x* (and other variables) can be displayed against estimated aridity *α* can be interpreted in dynamical terms. If we think of aridity as a time-dependent parameter, movement in the aridity axis triggers responses that can be seen as shifts in the (*α*, *x*) plot. The sample provides a solid set of predictions concerning the timing of these shifts worldwide.

Another well-known case study is provided by coral reefs, which have experienced significant declines worldwide (figure 1*d*). Here too, a perfect storm of nonlinear effects have changed these highly diverse ecosystems. A combination of human-dominated actions along with Allee effects, habitat loss and fragmentation along with pollution and overfishing and extreme events has been devastating [64,65]. Corals are intrinsically symbiotic, and that adds an extra nonlinearity associated with cooperative interactions^3^ between the coral animal and its algal companion (single-celled dinoflagellates). The large scale of bleaching events are a worrying signal of how the future can be. Because of our accurate understanding of temperature-related changes on the symbiotic pair, predictions are relatively easy to make. The example shown in figure 1*d* (centre) is just an illustration of a general trend, where corals might fail to adapt to a warming planet. Although they have traditionally been considered as resilient [67], the truth is that they have bleaching events that have killed coral across vast areas. Since bleaching events are becoming more frequent (at intervals of about 6 years), and since recovery from mass bleaching requires an estimated 10–15 years for fastest-growing corals, solutions in this case have a pressing time window as we approach 2050.

Large-scale modelling of the challenges associated with the biodiversity crisis requires sensing global, spatio-temporal complexity across scales [68], using meta community approaches [69,70], incorporating humans into ecology [71] and adopting a network perspective [72,73]. Concerning the first, our perception of the state of the planet has much improved over the last 30 years as remote sensing methods and machine learning-based data analysis have been developed. Some of these methods might soon allow us to tackle warning signals in very accurate ways while helping to identify the proper class of mathematical model describing the transition [74]. The recent incorporation of genomics can further help assessing and monitoring restoration efforts [75,76]. Since ecosystem degradation or even collapse is connected to loss of resilience, an ambitious goal would be to define a reliable sensing method that provides a measurable index [77,78]. Using as a working definition of resilience, namely the capacity of a system to recover from perturbations, Lenton *et al*. [68] have suggested a practical implementation. This idea stems from the dynamical correlations displayed by the behaviour of a system after perturbation. By monitoring ecosystems over a given time scale (associated with climatic fluctuations), the analysis of ecosystem changes (in space and time) would allow detection of shifts in resilience over time (although the data requirements might be demanding). Here, the combination of remote sensing methods with nonlinear dynamical systems makes it possible to inform several scales of biosphere governance and management.

In order to tackle these tipping points, including their detection and prevention, a major challenge of this research concerns their occurrence in time [72]. Although most models support the likelihood of these transitions (see however [79]) there is no general method that consistently predicts *when* the shift will occur. The problem has been tackled by defining so-called *warning signals* (WS), i.e. statistical patters of fluctuations that are expected to occur close to critical points [9,80]. This phenomenon is well known since the dawn of the theory of phase transitions [44,81,82]. These WS are characterized by the presence of long-range correlations both in time and space. They are quantitatively defined from the variance of ecological time series, but even a deterministic one-dimensional model helps to see where they come from. As an example, consider a simple model of habitat loss, such as [53]

3.5 $$\frac{dx}{dt}=F_{\mu }(x)=cx(1-D-x)-\delta x.$$

Here, as with equation (3.2), colonization (now linear) and extinction occur in a finite habitat. As before, the parameter set is *μ* = {*c*, *δ*, *D*}. It can be easily shown that a non-zero equilibrium point x* = 1 − *D* − *δ*/*c* exists provided that *D* < *D*_*c*_ = 1 − *δ*/*c*. The critical destruction level *D*_*c*_ separates population persistence from extinction. How is the system changing when approaching x* from a given initial condition *x*_0_? If we indicate by *y*(*t*) the distance to the fixed point x*, it can be shown [44] that

3.6 $$y(t)∼e^{-c(D_{c}-D)t}.$$

But this means that this distance will decay more and more slowly as *D* → *D*_*c*_. For *D* ∼ *D*_*c*_, this time will be infinite. This slow relaxation is known as *critical slowing down* and is a characteristic feature of continuous phase transitions [9,44].

Another necessary step towards a complex systems picture of the biosphere is to consider both humans and their environment altogether. This has been an ambition from the early days of ecological engineering [83]. The role played by humans in managing or disturbing ecosystems is well known in modelling hunting and fishing. Despite the obvious need for such integration, much is still to be developed, as discussed in Farahbakhsh *et al.* [71]. In our previous example concerning demographic explosions, the role played by social and cultural components was somewhat encapsulated in the carrying capacity. But an explicit consideration of socio-ecological feedbacks must take this into account. These Coupled Human and Environmental Systems (CHES) models are a major challenge, given the diverse nature of key processes that are to be included. Ideally, CHES models should allow us to understand the presence and nature of transitions between alternative states and inform us about WS while including social norms and learning [84]. In Farahbakhsh *et al.* [71], a replicator dynamics model is proposed as a theoretical framework, where

3.7 $$\frac{dx}{dt}=\sigma x(1-x)\Delta U(x,R)$$

is a dynamical system where *x* represents the relative fraction of the population adopting a conservation/mitigation option while exploiting a limited resource *R*. Conversely, 1 − *x* will be the rest of the population, which would adhere to a non-conservation policy. The last term, Δ*U*(*x*, *R*), introduces a difference between utilities [85]. Additionally, a dynamical equation for *R* (i.e. d*R*/d*t* = *ϕ*(*x* · *R*) is also used to introduce the specific effects of human actions on the environment. Although the standard response of such a resource to harvesting already includes sudden transitions, the presence of agents that make decisions and learn makes predictions more difficult due to the emergence of new potential states and transitions. More importantly, when conservation costs and resource abundance interact, one possible outcome of these models is a spontaneous evolution towards a regime shift [71,84]. This is in fact a crucial element of future CHES developments: allowing parameters to be part of the dynamical behaviour of the model.

## 4. Biodiversity, adaptation and engineering

**Figure 2.**
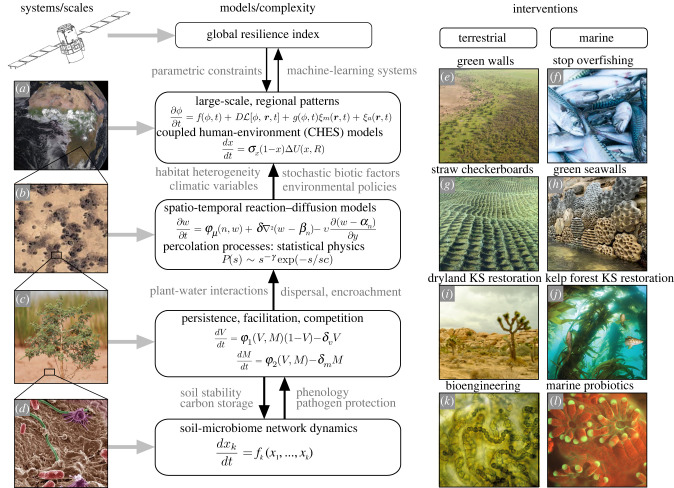
Scales, models and interventions. Our understanding of different patterns and processes in ecosystems, from molecules and cells to the global climate can be explored by a diverse range of mathematical models (central column). Each model addresses a given scale and is intended to answer specific questions that make sense on that scale. Here, we have used drylands as a case study. Four potential levels of study are: (*a*) large-scale dynamics taking place on the regional/continental level, where the social component might be needed; these models, along with remote sensing data and other sources of information, can help to define a global resilience index; (*b*) spatio-temporal processes associated with community dynamics involving facilitation; (*c*) species-level models introducing both low-dimensional pairwise exchanges and phenology; and (*d*) soil microbiome dynamics, where models can consider diverse levels of description (including multispecies equations). In the right column, four examples of interventions are indicated: (*e*) large-scale reforestation or afforestation, with the African Green Wall as one particularly relevant case study (image by UNCCD) aimed at creating a 7000 km long barrier; (*f*) implementing global policies to limit overfishing; (*g*) straw checkerboards used to allow planting of sand-binding vegetation in the Tengger desert leading to soil restoration (image from [86]); (*h*) green seawalls close to urban coastal areas.) Species-specific interventions can be designed for keystone species (KS). This is the case for Joshua trees (*i*) in drylands or kelp forests (*j*) in marine coastal communities. (*k*) Both restoration and bioengineering strategies can be developed by using cyanobacteria as key components of soil communities used to improve structural cohesion, enhance organic carbon and/or water storage. (*l*) Similar goals can be achieved by using synthetic microbiomes to increase resilience of corals.

As mentioned above, anthropogenic changes are taking place rapidly, leading to a shrinking window of opportunity. What is the ‘right’ scale for action? In previous sections, we have discussed several approximations involving monitoring biodiversity and potential WS. In this section, we will briefly review recent proposals associated with different ways of approaching ecosystem degradation and biodiversity losses under an intervention/engineering view. For each level, different mathematical and computational models are required (figure 2, using drylands as case study) and the presence of emergent properties is highlighted by the fact that to move from one level to another, new key components need to be incorporated that cannot be reduced to those used at the smaller scale. From top to bottom in figure 1*a*–*d*, we have (*a*) global drylands, (*b*) mesoscale landscapes (displaying pattern-forming phenomena), (*c*) individual phenology and pairwise plant–plant interactions and (*d*) the soil microbiome.

Each scale too is connected to different kinds of interventions, some of which are indicated in figure 2 for both terrestrial and marine ecosystems (righthand columns). In this case, large-scale examples of interventions would include the creation of green walls (*e*) or implementing common practices aiming at the reduction of overfishing (*f*). On a mesoscale, engineering habitats can include the building of straw checkerboards to freeze sand dune and restore soil crusts (*g*) or the building of seawalls in urban marine environments (*h*). Moving on the species-level, protecting and expanding populations of some keystone species such as Joshua trees (*i*) or kelp forest (*j*) can promote self-organization processes that foster biodiversity. Finally, on the smaller scale dealing with microbial populations, are bioengineering strategies involving cyanobacteria (*k*) or probiotics (*l*) aimed at restoring drylands and coral reefs, respectively. Are all these approaches feasible? What are their limitations?

Let us start with a bottom-up perspective to consider species-focused interventions. As pointed out in Lagerstrom *et al.* [87], we need to identify adaptive mechanisms that can be used by given species over the next few decades. Specifically, these authors suggest that the ability to respond and adapt to change—the so-called *adaptive capacity*—should be used as a guide for future decisions to help species and ecosystems to adjust to change. However, although some room for plasticity is known to be present (such as adaptation to drought in plants), most species have a limited genetic evolutionary potential to adapt on a few-decades time scale.

One of the big issues associated with the loss or introduction of keystone species is tied to network architecture. Any strategy aimed at protecting ecosystem resilience requires the identification of relevant species whose loss can trigger avalanches of change [72,88–91]. The potential for such downstream effects in food webs is illustrated by the effects of the loss of keystone predators in Barro Colorado Island [92] or in Yellowstone [87,93,94]. In both cases, along with the rapid changes in population numbers of herbivores (once the predator control is lost) multiple indirect effects are unleashed, affecting other species' abundances as well as habitat structure. In this case, the complex adaptive nature of ecological communities is associated with the presence of self-organization: we focus on a species that is known to foster biodiversity through its role as ecosystem engineer, whose effects propagate across ecological webs. These effects are exacerbated by climate-induced shifts in range of species [95]. In both marine and terrestrial ecosystems, changes in the distribution of life on Earth are affecting ecosystem health as well as human well-being. Within marine ecosystems, kelp forests (figure 2*j*) are a very important target for conservation and restoration that is also greatly threatened by diverse Anthropogenic stresses. It is now estimated that they cover no less than 28% of the world’s coastal areas. They are declining everywhere and that has enormous consequences given the essential ecosystem services that they provide. For some key species in terrestrial habitats, such as Joshua trees [87], habitat loss and warming can end in extinction by the end of the century unless active protection is implemented. But again, telling the time for extinction is not easy. An added complication in predicting extinction is connected to the existence of very long transients in ecosystem responses [96]. It is now well known that some dynamical systems close to catastrophic shifts can exhibit extremely long delays before they jump into collapse. This is the case for example of green-desert transitions [97,98] in drylands models, where vegetation cover might persist long after crossing the tipping point. Since a species can live in this transient configuration, it might appear healthy when in fact collapse is inevitable. There is however a bright side: unexpectedly, models also indicate that small perturbations could help maintain the ecosystem in the green phase [97].

How can we actively intervene to avoid biodiversity losses associated with climate change? The conservation and restoration strategies discussed in previous sections have in the past shown their potential to protect or enhance biodiversity. Will they be enough as we move through the twenty-first century? One controversial suggestion is the use of geoengineering strategies to mitigate the effects of climate change (see [99] and references cited). This climate engineering scheme operates on diverse physical or chemical factors. The cost of most proposed solutions is typically enormous, as a consequence of the massive scales involved and the risk of unexpected consequences. This includes a whole repertoire of proposals, from hundreds of thousands of towers to capture carbon dioxide to trillions of small, free-flying spacecrafts or ocean iron seeding and stratospheric aerosol injection. These solar geoengineering strategies [100] only influence warming by reducing solar insolation, and thus have no direct impact on increased CO_2_ levels. However, these alternatives remain on the table of potential pathways for mitigation in the long run after temperature overshoot occurs. This means a scenario where the 1.5–2° limits are transiently exceeded. But the urgency of avoiding critical values in global average temperature is illustrated by the analysis of long-term biodiversity trends. When this path is simulated using available data from 30 000 species (with given tolerance thresholds when exposed to warmer conditions) it is found that major damage to biodiversity will take place [101].

Since carbon removal is the highest priority, are there engineering approaches to address the problem? Several projects involving native tree planting in localized areas, often close to urban centres, improved air and water quality while helping carbon capture. Similarly, restoration efforts grounded in planting of sand-binding vegetation in drylands have proven effective to achieve soil crust rehabilitation. This is the case of the Tengger desert study [86,102] where an intensive engineering effort affected a 16 km long by 500 m wide area (figure 1). Further restoration efforts included planting shrubs once the sand surface was stabilized. This medium-scale restoration effort required a time scale for soil crust recovery of 30–50 years, thus consistent with the 2050 goals. Within the urban-related marine context, a very promising intervention deals with the use of eco-engineering (design for ecological co-benefits) marine urban structures [103–105]. By using a diverse range of sea walls with different habitat panels having water-retaining features (figure 2*h*), it has been shown that enhancing habitat heterogeneity in these otherwise featureless areas can boost biodiversity.

Moving into the regional/global scales requires dealing with strong constraints. Consider tree planting in habitats where no forests are present. When dealing with large-scale afforestation, the greatest obstacles emerge from the water requirements. Such projects have been shown to create undesirable effects due to increased runoff or reduction of water availability along with other drawbacks [106–108]. Big hopes have been focused for example in the creation of *green walls,* i.e. massive afforestation of millions of hectares. The African Green Wall (figure 2*e*) is one example of such megaengineering that aims at creating a living barrier to the expansion of the Sahara desert. Here too both human and climatic factors are equally relevant; and despite the potential promise, costs are high and climate and human factors interact [109]. A similar situation is to be found when dealing with large-scale management of marine ecosystems. In this case, losses are smaller than those seen in land ecosystems, but nevertheless the abundances of marine animals and habitats have been shrinking at an alarming rate. Among other measures, reductions in hunting pressure, the management of fisheries (figure 2*f*), along with habitat protection measures could allow a major rebuilding of marine life within the 2050 horizon [110]. However, this requires on the one hand a sustained commitment of financial resources and on the other the requirement that global warming is mitigated.

A less explored path involves the bioengineering of ecosystems by means of synthetic biology and other strategies aimed at the modification of genomes or microbiomes. This has been traditionally a controversial approach due to the concerns raised by the possible unintended consequences of manipulating organisms and in particular their delivery in natural environments. These concerns started to influence our thinking in the aftermath of recombinant DNA technology of the 1980s [111,112]. As a consequence, although their viability was known to be extremely limited when deployed in a field context, the potential use of genetically modified organisms (GMO) for field applications was banned in some places. Despite the complete lack of real tests, a general consensus was soon established: recombinant DNA should not be used because of potential unintended consequences. The situation has been slowly changing (with some reluctance) with the widespread use of engineered crops or the promise of mosquito-borne disease eradication by means of gene-drive technologies [113]. With the rise of synthetic biology and the use of genomics to sense and monitor biodiversity [75] or even transform agriculture [114], a new wave of possible designs and implementations has emerged, particularly within the context of microbiomes [115–117].

One particular proposal in this context is the idea of *terraforming* extant ecosystems [118]. Although the term has been originally used within the context of planetary engineering (see [119] and references therein), here the aim is to change communities that are or can be under threat of experiencing catastrophic shifts. This approach strongly departs from the simplistic GMO deployment picture and goes far beyond standard bioremediation scales [120–122]. A crucial difference is the systems view taken, with two main goals: introduce some functional trait that improves systems-level properties while biodiversity is preserved (or even enhanced). The design principles also aim at a control of the engineered strains thanks to ecological nonlinearities. In this context, some ecological interaction motifs [123] and network-level constraints [124] act as firewalls to the spread of synthetic microbes. An example of this view is the potential of terraforming for drylands [97,98,125]. In this case, species that play a key role in maintaining soil integrity and organic carbon levels, such as cyanobacteria (figure 2*k*), would be used as engineering targets. Adding an extra function such as secreting a polymer that can enhance water retention, even at low levels, could generate a systems-level improvement and move away the location of an undesirable ecological shift. In general, the potential of microbial biotechnology could be expanded across all the scales outlined in figure 2, with microorganisms at the core of novel strategies [126]. The idea that synthetic biology can be a helpful (and even necessary) tool to protect biodiversity has been further actively discussed in conservation goals [75,113] within marine biology. Two examples are provided by kelp forests [127] and coral reefs [128] which play keystone roles in their habitats. In both cases, engineering their microbiomes could be the right strategy to promote their recovery from damaging events (such as habitat degradation or bleaching).

Much research is needed here, since little is known about the potential success of modifying ecosystems by means of species-level engineering. But one case study might indirectly support the success of the strategy. The study of oceanic plastic debris has shown that the total amount of plastic waste measured in the marine environment is much less than expected from estimated deployment rates, often not showing a growing trend. A resource–consumer model of plastic–microbiome interactions predicts that, under the presence of plastic-degrading microbes, a characteristic stabilization of surface plastic would be expected while the population of these microbes would increase in proportion to the rate of plastic deployment [129]. Recent global metagenomic analyses have confirmed those predictions. Plastic-degrading microbes indeed are widespread and might account for reduced plastic abundance [130] and abundances appear positively correlated with pollution trends [131]. This indicates that evolved microbial populations can develop and perform efficient bioremediation tasks (without ecological disruptions) within a time window of decades.

## 5. Discussion

A general consensus among scientists from very diverse disciplines has been emerging about the future of our planet and our society. As global warming and an intensive exploitation of planet resources keeps rapidly increasing, WS indicate that potentially catastrophic transitions will unfold within this century. Wide weather fluctuations, alarming biodiversity declines and social unrest are already here. Predicted climate change scenarios seem confirmed and consistent with worst-case outcomes. What can be done to reverse, counterbalance or prevent tipping points? Many different solutions have been suggested based on sustainable growth, restoration strategies and increased clean energy use. But the time scale for effective measures is rapidly shrinking. Confronted with a planet decline where humans are part of a complex, endangered ecological network, novel approaches need to be taken. All these approaches include unsolved, multiscale problems and will need to be applied in a social context dominated by cities, political instability and rising inequality. A complex systems perspective including all key faces of the problem is required, pointing to an agenda of well-defined alternatives. Changing ecosystems, either following bottom-up (synthetic biology) or top-down (afforestation, geoengineering) approximations needs to be carefully considered, and different strategies are compatible. What is the optimal way of bringing together biodiversity and human interests? As pointed out by Howard Odum, any ecological engineering approach needs to join human design and environmental self-design so that they are mutually symbiotic [83]. To make this a reality, preserving and fostering biodiversity is a necessary condition.

How can we know for sure the state of the biosphere by 2050? Can our ambition of an accurate prediction be fulfilled? As shown by the success of climate science, predictions are not only possible but essential to define strategic mitigation and adaptation roadmaps. The diverse range of proposals discussed here span a range of views that needs to be used as a source of alternative, but complementary solutions. We cannot yet know if 2050 will be characterized by the success of large-scale protection or instead will be (as pointed to in [87]) dominated by novel ecosystems. As pointed out by the physicist Denis Gabor, predicting the future might be difficult, but we can also think out of the box. Our species has been a too successful ecosystem engineer, transforming a planet where ecosystems are nowadays being dismantled. We face an uncertain future with limited resources exploited by a fast-growing human population and where biodiversity needs to be protected. Biodiversity is central in providing society with the required goods and services to sustain itself [132]. Action is needed to preserve it while ensuring the well-being of humans.

Any future solution will necessarily involve considering the whole range of strategies described by the different contributions of this theme issue. Science will also need citizen awareness of the problems involved and a proper governance. As we write this paper, humanity is moving out from a 2-year pandemic event that is a reminder of the global nature of the Anthropocene [133]. Dealing with COVID-19 required an enormous collective scientific action that ended up with effective vaccines in a very short time window. But it has also revealed our weaknesses. The reality of climate change and its consequences are upon us and global decisions will be needed again. The complex, nonlinear nature of our biosphere makes it difficult to design simple solutions. New ideas and integrative strategies involving multiple scales will be needed as we keep pushing our understanding of this unique planet and reconsider our future place as a node of the living web.

## Data Availability

This article has no additional data.

## Footnotes

1 This can be generalized by considering a noise term that is *multiplicative*, i. e. We have d*x*/d*t* = *f*_*μ*_(*x*) + *g*(*x*)*ξ*(*t*) where *g*(*x*) is an extra function that modulates the noise by means of a coupling with population size. In this case, noise can play a new role and even generate attractors that are not present in the deterministic (noise-free) model. These phenomena might have a specially relevant role in environmental systems, in particular, in relation to drylands [46].

2 Formally, this popular representation is obtained, for one-dimensional systems, from the so-called potential function. The dynamical system is said to *derive* from a potential V(S) [44,61,62] if we can write it as

3.4 $$\frac{dx}{dt}=-\frac{dV}{dx},$$

i.e. when the changes in the state of the system obey a *gradient response*: the steeper the derivative in the right-hand side, the larger the damping in the opposite direction. For a noise-free one-dimensional system, this function simply reads $V(x)=-\int f_{\mu }(x)\;dx$. It can be shown that the minima (maxima) of V(x) correspond to the stable (unstable) fixed points.

3 Here, the cooperative loop results in fact from a reciprocally selfish exchange. As discussed in Herre *et al.* [66], other things being equal, each component of the pair will tend to maximize its net gain from the coral–algae exchange (by minimizing costs and maximizing benefits). This selfishness has the potential to lead to ecological instability.
